# Twenty years of emotional-behavioral problems of community adolescents living in Italy measured through the Achenbach system of empirically based assessment (ASEBA): a systematic review and meta-analysis

**DOI:** 10.3389/fpsyt.2023.1161917

**Published:** 2023-12-11

**Authors:** Cecilia Serena Pace, Stefania Muzi, Alessandra Frigerio, Wanda Morganti, Victoria Bianchi, Guyonne Rogier

**Affiliations:** ^1^Department of Education Sciences, School of Social Sciences, University of Genoa, Genova, Italy; ^2^Scientific Institute, IRCCS E. Medea, Child Psychopathology Unit, Bosisio Parini, Lecco, Italy

**Keywords:** internalizing problems, externalizing problems, child behavior checklist 6–18 (CBCL 6–18), youth self-report 11–18 (YSR 11–18), adolescents

## Abstract

**Background:**

This is a systematic review and meta-analysis of emotional and behavioral problems among Italian community adolescents in the last 20 years, as assessed through the ASEBA questionnaires CBCL 6–18, YSR 11–18 and TRF 6–18. Research questions address: (1) pooled means of problems’ scores in questionnaires scales; (2–3) variations in scores according to sociodemographic and time-related factors, and studies’ quality; (4) trends in research with ASEBA instruments along with other outcomes, e.g., psychopathological symptoms.

**Methods:**

A systematic literature review of Scopus, EBSCO, PubMed, Web of Science, and ProQuest databases using the PRISMA 2020 guidelines was conducted on November, 2021, and of grey literature on December, 2021. The quality of studies was assessed through the Newcastle-Ottawa Scale.

**Results:**

Forty-four studies were eligible for the systematic review, of which 34 were included for meta-analysis. Results showed that: (1) emotional-behavioral problems were higher when assessed by the CBCL and lower when assessed by the YSR compared to normative data; (2) there were no gender and age differences, except for higher scores of Anxious/Depression symptoms, in girls. (3) internalizing and attention problems increased over the last two decades. (4) major trends of Italian research investigate adolescents’ emotional behavioral problems concerning attachment, comorbid symptoms, especially internet addictions, and eating disorders.

**Discussion:**

Despite some limitations (e.g., low-medium quality of most studies, no data on the TRF, under-representation of some geographical areas, some search-related choices), these data provides Italian practitioners and international researchers of some parameter to evaluate Italian adolescents emotional-behavioral problems. Registered on PROSPERO N. CRD42022299999.

## Introduction

The World Health Organization defines “adolescence” as the period of life between 10 to 19 years old (1), indicating it as a time of developmental risk for the onset of mental disorders (1), which could be predictive of poor social and health adjustment up to adulthood (2). Therefore, the World Mental Health [WHO] encourages research on prodromal signs, developmental processes, and variations of adolescent mental health disorders, useful to support prevention as a priority (1). In this regard, decades of research have established the preventive utility of early detection of prodromic symptoms of mental disorders in the form of “*emotional-behavioral problems”* (1, 3, 4). According to Achenbach’s definition (3), these include *internalizing problems* – e.g., anxiety, depression, and/or withdrawal - and *externalizing problems*, such as behavioral problems (3), as well as other types of typical symptoms found in adolescence. These include namely *social problems* (like shyness, bullying, substance, and alcohol use or abuse), *thought problems* including dissociative symptoms, and *attention problems* (3).

Recent data show an increase in emotional-behavioral problems among adolescents in the last decades (5), and even more because of the COVID-19 pandemic (6). Specifically, pre- and post-pandemic evidence suggests an increase in anxiety and depression, particularly in girls (5, 7, 8). The last systematic review on the topic – dated 2014 – reports no increase in externalizing difficulties (9). This in apparent contrast with later contributions which show a rising prevalence of conduct disorders in clinical settings (7). Moreover, pandemic studies reveal contrasting findings. They either occasionally document no change (8) or show an increase in externalizing difficulties during the COVID-19 pandemic (10), especially when subclinical behavioral symptoms were present before the disease’s outbreak (11). Therefore, updating the meta-analytical data on the levels of adolescent emotional-behavioral difficulties may help to understand how they have varied over the last decade, including the pandemic years (12). This supports the research and prevention goals defined by the WHO (1).

An update appears crucial for Italy, where the latest epidemiological data date back to more than 10 years ago (13, 14). According to this information, Italy fell into average European values at that time (13, 14), with a prevalence of total problems around 8.2%. However, a recent UNICEF report estimates that 16.6% of Italian adolescents experienced a mental health condition in 2019, with a European prevalence of 19%, twice as much as 10 years ago (15). Therefore, updating data on the current state of Italy may help to understand if emotional-behavioral problems have increased. Moreover, it may show whether existing subthreshold problems are aggravated until the criteria for a psychiatric diagnosis are met, which types of problems have remained stable, and which have changed.

For this purpose, four decades of research depict the Achenbach Empirically Based Assessment System (ASEBA) (16, 17) as a reliable and widely used method of assessment for internalizing and externalizing difficulties in the age range of 6–18 years. This system is translated into more than one hundred languages and used in both research and clinical settings (4). Indeed, a recent review on internalizing and externalizing difficulties in children (4) found that 554 of 592 studies employed the ASEBA instruments. To date, cross-cultural comparisons with the ASEBA system greatly contributed to understanding trends in adolescent mental health disorders, for example by detecting more internalizing problems in girls and externalizing and attentional problems in boys, and higher syndrome scale scores in older teenagers (18).

Specifically, the system comprises three parallel questionnaires that can be used with adolescents: The parent-report Child Behavior Checklist (CBCL), the self-report Youth Self Report (YSR), and the teacher-report Teacher Report Form (TRF). After continuous empirically based modifications and updates in the items, (19), the latest versions of these questionnaires are dated back to 2001, specifically the CBCL 6–18 years, YSR 11–18 years, and TRF 6–18 years. All three are composed of a first part with questions on adaptive functioning and a second part that assesses emotional-behavioral problems through 113 items on a 3-point Likert scale. These questionnaires evaluate children’s problems according to 8 syndrome scales and three broadband scales. The broadband *Total problems* scale is the sum of all items; the *Internalizing problems* scale includes syndrome scales *Withdrawn/Depressed, Anxious/Depressed,* and *Somatic Complaints*; the *Externalizing problems* scale sums scores of *Aggressive Behavior* and *Rule-breaking* (CBCL 6–18)/*Delinquent Behavior* (YSR) syndromes scales. In addition, there are other three syndrome scales for *Social problems, Attention problems,* and *Thought problems*.

The ASEBA questionnaires can also be used to support a diagnosis based on criteria of the more recent version of the Diagnostic and Statistical Manual of mental disorders (DSM-5) (3). The DSM-oriented scales for the age range 6–18 refer to *Affective problems, Anxiety problems, Somatic problems, Attention Deficit/Hyperactivity problems, Oppositional-Defiant problems,* and *Conduct problems* (20). However, these have not been considered in this study as poorly used with non-clinical populations, and generally less employed compared to scores (16).

In Italy, the largest and most well-known study which used the 2001 version of the ASEBA is epidemiological research dating back to 2009 (21). However, the only available normative data comes from the previous versions of the CBCL and the TRF, which are dated back to 1991. These instruments show reliable psychometric properties are extensively used for both research and clinical purposes in the Italian population (13). Therefore, the current systematic review and meta-analysis focused on studies where the emotional-behavioral difficulties of Italian adolescents have been assessed through the CBCL, YSR, and TRF. The aim is to contribute to an update of the current knowledge of Italian adolescents’ mental health. To ensure grasping eventual changes in the levels of emotional-behavioral difficulties over time, this review included the versions of the questionnaires released in 2001, which are the most used in the last 20 years, especially in the last decade. Gender and age differences were also considered to further explore similarities and discrepancies with previous literature. Moreover, the ASEBA research has also identified connections between emotional-behavioral difficulties and other outcomes, such as comorbid “new” symptoms [e.g., internet addiction (22)], or psychological (e.g., attachment) or biographical (e.g., exposure to childhood adversities) characteristics (23, 24). For this reason, this review additionally aims to identify major trends in research on emotional-behavioral difficulties and other outcomes, to highlight possible foci of future meta-analyses. This will provide useful information to compare with data retrieved from the ASEBA questionnaires in populations that will be the object of a second part of this review. Specifically, these are clinical populations of adolescents who have received a diagnosis for a mental health disorder according to criteria of the fifth version of the Diagnostic and Statistical Manual of Mental Disorders (3) or the International Statistical Classification of Diseases, Injuries and Causes of Death version 11 (25). There are also adolescents at risk for the development of mental health disorders because of socioeconomic disadvantage (26), medical disorders, e.g., diabetes (27), or unfavorable biographic experiences, e.g., exposure to disaster or early placement in adoption, foster care or residential care due to childhood adversities (28).

Lastly, the methodological characteristics and quality of the studies will be reviewed and evaluated to assess their impact on the reported estimation of emotional and behavioral difficulties of Italian adolescents. This will help readers to frame the results by identifying the strengths and weaknesses of the current research and to formulate suggestions directing future research.

## Objectives

This study aims to answer four research questions:

1) RQ1 What were the pooled mean scores of Italian adolescents’ emotional-behavioral problems -in terms of total, externalizing, internalizing problems, and specific scales- assessed through ASEBA?
2) RQ2 Do scores of emotional-behavioral problems vary according to socio-demographic (i.e., gender and age) variables?
3) RQ3 Were there any changes in problems’ scores over 20 years? And after the COVID-19 pandemic? Do the scores of emotional-behavioral problems vary according to methodological characteristics and the quality of the studies?
4) RQ4 What are the major trends in the research on relationships between emotional-behavioral difficulties of Italian adolescents and other outcomes, in terms of comorbid symptoms, or psychological and biographical features?

## Methods

### Protocol registration

The format of the methods and results was based on the Preferred Reporting Items for Systematic Reviews and Meta-Analyses (PRISMA) 2020 guidelines (29). The study was pre-registered on PROSPERO (No. CRD42022299999).

### Eligibility criteria

Inclusion and exclusion criteria are summarized below according to the PICOS format, except for the Comparison criteria which was not relevant for the aims of this systematic review.

- Population: Community adolescents aged 11–18 years living in Italy and without a psychiatric diagnosis or adolescents at-risk for a psychiatric disorder as defined in the introduction (3, 25–28). The latter population will be analyzed in the second part of this review.
- Intervention: Administration of at least one of the ASEBA instruments of the latest version from 2001 (i.e., CBCL 6–18, YSR 11–18, and TRF 6–18).
- Outcomes: The raw mean obtained by participants in at least one syndrome and/or broadband scale. The corresponding author was contacted when this information was not provided (i.e., not available in the full text of the article or full text not retrieved). In case this was not available, the contribution was excluded from meta-analyses but still remained eligible for the qualitative review.
- Study: Empirical and quantitative studies with original and not overlapping data, including gray literature.

### Search strategy

#### Information sources

Searches were performed via Scopus, EBSCO (PsycINFO, PsycArticles and Behavioral Science Collection), PubMed, and all databases of Web of Science and ProQuest (listed in Appendix A). Gray literature was searched through the following strategies: checking the first 200 records on Google Scholar (30) asking for unpublished data from the contacted authors and sharing the unpublished data of team member Alessandra Frigerio. The latter required the stipulation of a registered agreement between the University of Genoa and the Scientific Institute *E. Medea*. Moreover, to complete the whole search strategy, a cross-check on the reference lists of the included contributions was performed. Searches on academic databases were performed on November 22nd, 2021, and the search for gray literature was performed on December 16th, 2021.

#### Search strategy

To retrieve contributions, sources were identified and keywords were listed to create a syntax of operationalized research questions. Keywords corresponded to two main constructs, namely “ASEBA” and “Italian,” related through the Boolean operator AND. Then, this list (detailed in Appendix A) was adapted to the respective languages of databases. A reduced syntax was used to search for gray literature on Google Scholar (see Appendix A).

#### Selection process

**Figure 1:**
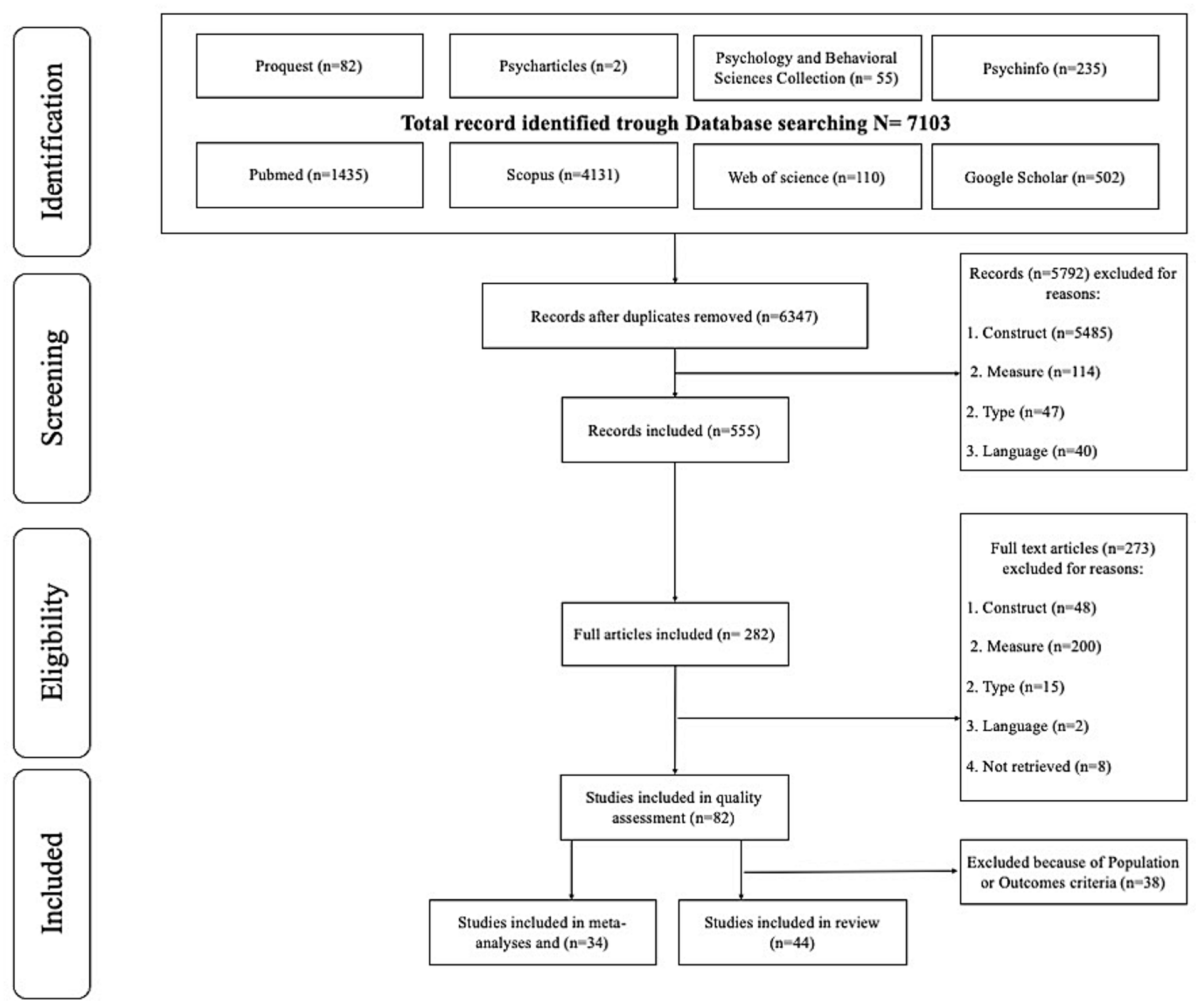
Flow diagram summarizing the identification and selection process.

Following the PRISMA 2020 guidelines (29) and using the Zotero software, duplicates were removed. Following this operation, from 7,103 records, 6,347 remained for evaluation. Subsequently, two authors (WM, VB) independently screened abstracts and titles of the records through Zotero^©^ Software, according to inclusion and exclusion criteria. After this screening process, full texts of the included records (*n* = 555) were downloaded and screened for eligibility in line with the inclusion and exclusion criteria. Disagreements in each phase were discussed and resolved by consensus (inter-rater agreement rate 92.73%). The selection process led to the final inclusion of 44 full texts including data on community adolescents. This is illustrated in Figure 1. Data on at-risk and clinical populations of adolescents will be the focus of the second part of this meta-analytic review. By the end of this procedure, all 44 full texts were eligible for the qualitative review but only 34 were eligible for meta-analyses.

### Data extraction

Two independent researchers (WM, VB) carried out the data extraction. Any arising discrepancies were resolved through consensus, consulting a third researcher (GR) if an agreement was not attained. For each contribution, the following data were extracted: (i) *characteristics of the contributions*: Authors, Publication year (coded as 2022 – publication year), publication status (published versus unpublished), diffusion (published in an international versus Italian journal); (ii) *characteristics of participants*: sample size (for both males and females, only males, and only females), gender composition (coded as % of males), mean age (for both males and females, only males, and only females), age range (in years); (iii) *characteristics of methods*: research design (e.g., cross-sectional, experimental, longitudinal), ASEBA measure used/extracted (CBCL, YSR, or TRF), time of data collection in respect to the COVID-19 pandemic (pre or post-pandemic), quality assessment (see below); (iv) *outcomes*: ASEBA scales (e.g., total, internalizing, externalizing), raw scores and standard deviations (for both males and females, only males, and only females).

### Quality assessment

Quality assessment for studies included in the meta-analyses was performed through an adapted version of the checklist Newcastle-Ottawa Scale [NOS; (31–33)] for epidemiological studies. This evaluates specific aspects regarding selection (e.g., definition and representativeness of the cases), comparability (related to the inclusion of confounders), and outcome (linked to criteria such as the quality of the measurement process and the registration of response rate), and then provides a total score of assessment quality. Authors WM and VB independently rated quality, with GR and SM solving disagreements.

### Statistical analyses

To compute pooled means, the *meta* and the *metafor* packages of the R software for Mac were used. These packages employed untransformed raw scores and account for the weight of the sample size to compute weighted pooled means (34). The random effect model was applied, according to the possibility that each study has an independent effect related to its sample (35). Also, when observations are significantly heterogeneous, random-effects models are thought to be more conservative and appropriate (36) allowing to make inferences regarding the general population.

In addition to the computation of pooled means, their standard errors, and their respective confidence interval (95%) to evaluate quality, the heterogeneity was explored using the *Q* statistic. Lastly, the moderating roles of continuous variables (gender composition, age, quality of the study, publication year) were assessed throughout the computation of meta-regression on pooled means and the test of heterogeneity throughout the *Q* statistic (33, 37). Because of the high homogeneity regarding categorical moderators (pre/post-pandemic period of data collection; design of research) and the consequent low statistical power, moderation analyses with categorical factors were not directly tested (38).

Instead, an exploratory approach was adopted performing sensitivity analyses. Indeed, when the number of studies with a value of a categorical moderator was low (i.e., ≤ 2) these contributions were left out and the changes in pooled mean, its statistical significance, and in the heterogeneity were evaluated. To better estimate the proportion of changes of these indexes from their original values, a percentage was computed. The same approach was adopted (i.e., sensitivity analyses) observing the changes in statistical indexes when leaving out studies with a small sample size (i.e., ≤ 25). The analyses were computed to check that studies with small sample sizes may significantly distort the estimation of average effect sizes.

Lastly, to estimate publication bias, funnel plots were created, visually inspected and the Egger linear regression method was used (39). In case of a statistically significant result (i.e., *p* < 0.01), a corrected effect size was calculated adopting Duval and Tweedie’s trim-and-fill method (40).

## Results

### Main characteristics of the included studies

The systematic search led to the identification of 44 independent contributions, and their main characteristics are displayed in Table 1. These studies were published between 2009 and 2021 and only one of them was unpublished (i.e., retrieved from grey literature search). Most were from international journals and only one was found in an Italian journal. Regarding the study design, three were experimental, one was longitudinal, and all of the others were cross-sectional. Sample sizes ranged from 13 to 3,399 participants and a total of 18,955 participants were on average between 11.12 and 17.40 years old. Most of the studies were conducted on mixed-gender samples, except for three which were carried out only among females and one only among males. No more than three researches were conducted after the COVID-19 pandemic. Then, 27 of the 44 studies reported data regarding the YSR, 17 of the CBCL 6–18, 4 of both YSR and CBCL, and none concerning the TRF. Lastly, concerning the risk of bias of the contributions included in the meta-analysis, results of the quality assessment evidenced 19.35% of studies were classified as high quality and the remaining as medium quality regarding the selection criteria. Regarding the exposure criteria, less satisfactory results were obtained, with 22.58% of studies being classified as “low.” Details of the total scores of quality assessments are available in Table 1.

**Table 1:** Main characteristics of the included studies (*N* = 44).

Study	Design	Tool	Sample						Pre/post pandemic	Certainty	Relationships with other variables
			N^a^	Coverage^b^	Gender	Age					
					% Males	Range	Mean	SD			
^*^Battistutta et al., 2009 (41)	Cross-sectional	YSR	135	Local, NE	34.1	11–18	13.9	1.4	Pre	2	N/A
^*^Bizzi, 2019^c^ (42)	Cross-sectional	CBCL	31	Local, NW	48.37	11–15	12.9	1.39	Pre	2	*Attachment* security dimensions are related to lower somatic complaints
^*^Bizzi and Pace, 2019 (43)	Cross-sectional	CBCL	32	Local, NW	46.88	11–12	12	1.39	Pre	3	N/A
^*^Bizzi et al., 2020 (44)	Cross-sectional	CBCL	69	2 cities NW, C	59.42	11–12	11.68	0.63	Pre	3	*Attachment* coherence are related to lower internalizing problems. *Reflective functioning* is related to lower internalizing and externalizing problems. *Verbal skills* are not related to problems.
^*^Bizzi et al., 2021 (45)	Cross-sectional	CBCL	70	Local, NW	69.01	11–13	12.14	0.97	Pre	2	*Attachment* secure or insecure classification is used as a parameter to compare clinical and community teens on symptoms.
Calderoni et al., 2015 (46)	Cross-sectional	YSR	50	C	0	11–18	14.30	1.85	Pre	–	Higher *autistic traits* are related to clinical levels of internalizing problems, i.e., exceeding clinical cut-off.
^*^Calvo et al., 2015 (47)	Cross-sectional	YSR	14	2 cities, NE, SE	71.43	–	–	–	Pre	1	N/A
Cerruti et al., 2017 (48)	Cross-sectional	YSR	240	C	48.1	11–15	11.8	0.97	Pre	–	Adolescents with higher scores of *internet addiction* (IA) show higher scores of total, internalizing and externalizing problems in the YSR. Higher IA predicts higher levels of internalizing and externalizing problems.
^*^Chiesi et al., 2017 (49)	Cross-sectional	YSR	662	C	54.1	11–18	13.87	2.17	Pre	2	Higher *mindfulness* is related to less depressive and attention problems.
^*^Cimino et al., 2021 (50)	Cross-sectional	YSR	739	C	51	–	13.4	1.2	Post	3	Higher *social media addiction* and less *perceived self-efficacy* are related to more total problems.
^*^Costabile et al., 2020 (51)	Cross-sectional	YSR	328	Regional, SW	54.7	14–18	16.67	1.50	–	1	*Social disconnectedness, Perceived illegitimacy of authorities, Radicalism, and Activism* are explored in relation to a composite score including also YSR items.
Costantino et al., 2011 (52)	Cross-sectional	YSR	42	NE	50	14–16	14.6	0.66	Pre	–	Adolescents classified as insecure in *attachment* show higher scores of internalizing problems, anxious/depressed, withdrawn, somatic complaints.
^*^Crescentini et al., 2020 (53)	Cross-sectional	CBCL	–	Multicentre, NE, C	49.7	11–18	12.54	1.50	Post	1	N/A
^*^De Santis et al., 2019 (54)	Cross-sectional	YSR	391	Multicentre, NE, SWI	30.0	14–18	15.91	2.02	Pre	3	*Attachment* dimensions of security are related to higher depression and lower anxiety, somatic complaints, rule-breaking, and aggressive behaviors. Higher problems in all these scales are related to attachment insecurity dimensions (avoidance or preoccupation).
^*^Feo et al., 2014 (55)	Experimental	YSR	342	Regional, C	–	11–14	–	–	Pre	3	N/A
^*^Frigerio et al., 2009^d^ (21)	Experimental	CBCL	3,399	Multicentre, NW, C, SWI	50.3	11–14	–	–	Pre	4	N/A
^*^Frigerio et al., 2019^d^ (56)	Cross-sectional	CBCL	494	/	40.7	11–18	14.86	2.41	Pre	–	N/A
Gatta et al., 2012 (57)	Cross-sectional	YSR	661	NE	70.3	14–18	14.90	1.60	Pre	–	Adolescents who *drink more alcohol* meet criteria for a diagnosis for an externalizing disorder (25.7%) or an internalizing disorder (15.2%) according to YSR DSM-oriented scales.
Guidetti et al., 2017 (58)	Cross-sectional	YSR	2,961	NW	48.16	11–13	12.27	0.98	Pre	–	Dimensions of *school affiliation* such as higher affiliation with teacher, bond with school, intrinsic motivation and positive attitude toward school, as well as lower dissatisfaction with teacher and negative emotionality toward school, are related to lower internalizing and externalizing problems.
^*^Gugliandolo et al., 2015 (59)	Cross-sectional	CBCL	263	Local, SWI	50.6	13–17	–	–	Pre	2	N/A
Kapetanovic et al., 2020 (60)	Longitudinal	YSR	194	Multicentre, C, SW		13–15	–	–	Pre	–	Patterns of *communication*. Higher adolescents’ secrecy predicts higher externalizing problems, while higher parental control are related to higher internalizing problems.
^*^Lisi, 2019 (61)	Cross-sectional	YSR	1,400	Regional, C	38.61	14–18	16	1.42	Pre	3	Higher both internalizing and externalizing problems are related to higher *psychological control* by the mother and father and lower *emotional intelligence*, *wellbeing, self-control, emotionality, sociality, self-motivation, and adaptability*.
^*^Malagoli and Usai, 2018 (62)	Cross-sectional	YSR	193	Local, NW	35.23	–	–	–	Pre	2	*Emotion dysregulation* dimensions are related to more social complaints and social problems. No relationships with working memory
^*^Malagoli, 2020 (63)	Experimental	YSR	125	2 cities, North	0.00	13–18	17.40	1.20	Pre	1	N/A
^*^Manna, 2020 (64)	Cross-sectional	YSR	387	Regional, SWI	56.6	13–18	15.75	1.52	Post	4	*Attachment* insecure-avoidant pattern is related to higher internalizing problems. According to the type of child maltreatment, insecure-dismissing and insecure-preoccupied individuals show more externalizing problems.
^*^Mascheretti et al., 2015 (65)	Cross-sectional	CBCL	13	2 cities, NW	53.8	11	11.12	11.1	Pre	2	N/A
Medda et al., 2019 (66)	Cross-sectional	YSR CBCL	382	–	56.3	14–18	16.37	1.22	Pre	–	Higher *sleep problems* are associated to lower effortful control, as a dimension related to higher attentional problems.
^*^Muzi et al., 2021 (67)	Cross-sectional	YSR	62	Regional, NW	37	12–17	15.43	1.65	Post	3	Higher *social media addiction* is associated with higher delinquency, thought, and social problems. Higher *attachment disorganization* is associated with more social problems
^*^Nobile et al., 2014 (68)	Longitudinal	CBCL	287	Regional, NW	50.9	–	12.09	0.89	Pre	4	N/A
^*^Operto et al., 2018 (69)	Cross-sectional	CBCL	23	Local, SE	39	11–18	14.75	3.17	Pre	2	Dimensions of higher *parenting stress* are not related to problems.
Oppo et al., 2019 (70)	Cross-sectional	YSR	1,336	Multicenter, N, C, S	42.1	11–18	14.46	2.16	Pre	–	Adolescents with lower *mindfulness skills* and higher *psychological inflexibility* show higher depressive symptoms in the YSR and CBCL. Higher social withdrawal is associated only with lower mindfulness skills, while higher somatic complaints only with higher psychological inflexibility.
^*^Pace and Muzi, 2019 (71)	Cross-sectional	YSR	382	Regional, NW	59	13–18	15.59	1.1	Pre	2	Higher *binge eating disorder* symptoms are predicted by either higher internalizing, externalizing, or other problems.
^*^Pace et al., 2020 (72)	Cross-sectional	CBCL	110	Regional, NW	50	11–17	14.22	1.84	Pre	4	*Attachment* dimensions of lower self-regard and higher rivalry toward sibling(s) are related to more internalizing problems and only the rivalry with more externalizing ones.
^*^Raffagnato et al., 2020 (73)	Cross-sectional	YSR	234	Local, NE	35.4	13–19	15.8	1.35	Pre	3	N/A
^*^Riva et al., 2015 (74)	Cross-sectional	CBCL	631	2 cities, NW	49.76	11–14	11.89	0.90	Pre	4	N/A
^*^Rothenberg et al., 2020 (75)	Cross-sectional	YSR	194	Local, C	50	12	12	–	Pre	3	*Emotion dysregulation* of sadness and anger predict higher depressive symptoms and aggressive behaviors, respectively.
^*^Scaini et al., 2021 (76)	Cross-sectional	YSR	744	2 cities, NW	51.6	–	15.73	1.23	–	3	N/A
Schweiger et al., 2017 (77)	Cross-sectional	YSR	644	Multicenter, N, C, S	41.1	11–18	14	2.05	Pre	–	Higher *psychological inflexibility*, resulting by higher cognitive fusion and experience avoidance, is related to higher total, internalizing and externalizing problems in the YSR.
Spatola et al., 2010 (78)	Cross-sectional	CBCL	–	N	–	12–17	–	–	Pre	–	N/A
^*^Tagliabue et al., 2018 (79)	Retrospective	YSR	301	Local, NW	43.23	16–18	17.00	–	Pre	1	Higher internalizing and externalizing problems are related to less authoritative and higher authoritarian *parenting styles*.
^*^Thommessen et al., 2013 (80)	Cross-sectional	CBCL	60	Local, C	100	–	17.14	0.35	Pre	2	N/A
^*^Troncone et al., 2020 (81)	Cross-sectional	CBCL	127	Local, SW	51	13–18	–	–	Pre	4	Higher internalizing problems are related to higher symptoms of *bulimia*; higher externalizing problems are related to higher *diabetes-related eating disordered* symptoms.
^*^Urgesi et al., 2012 (82)	Cross-sectional	CBCL	15	Local, NE	0	13–17	15.40	1.20	Pre	2	N/A
^*^Vismara et al., 2019 (83)	Cross-sectional	YSR	185	Regional, SWI	40	14–18	16.20	1.20	Pre	4	*Parental depressive symptoms* of mothers are related to both adolescents’ internalizing and externalizing problems (and subscales), of the fathers were related to internalizing ones (and subscales).

–, information not retrieved. N/A, Not applicable.

^a^The sum of participants in all studies is 18,955 Italian teenagers aged 11–19 years. ^b^area of data collection. Multicentre, more than one center in at least two regions. NE, North East, NW, North West, C, center, SE, South East, SW, South West (includes islands Sardinia and Sicily, marked with SWI). ^c^Unique study published in an Italian journal; ^d^Unpublished data; ^*^Included in meta-analyses.

### RQ1: the distribution of emotional behavioral problems

The number of studies, number of participants, and pooled means are shown in Table 2. Results are displayed for the total sample and separately for males and females.

**Table 2:** Pooled means of emotional-behavioral problems in the child behavior checklist 6–18^a^ and youth self-report 11–18 among Italian teenagers.

Dimension		Total sample				Males sample				Females sample			
CBCL^a^	Gend	k	N	Mean	95% CI	k	N	Mean	95% CI	k	N	Mean	95% CI
Total problems	50.01	11	4,645	31.20	[25.65; 36.75]	9	2,258	29.34	[23.82; 34.86]	8	2,260	31.13	[22.86; 39.38]
Internalizing problems	50.34	11	4,692	8.83	[7.22; 10.43]	11	2,297	8.85	[6.87; 10.82]	10	2,268	8.58	[7.08; 10.08]
Withdrawn/depressed	50.31	10	4,405	2.51	[2.13; 2.89]	9	2,151	2.51	[2.03; 2.97]	8	2,127	2.10	[1.80; 2.40]
Anxious/depressed	43.87	11	5,051	8.12	[6.21; 10.02]	9	2,151	3.34	[2.51; 4.17]	10	2,820	8.44	[5.84; 11.04]
Somatic complaints	50.25	11	5,051	2.36	[1.86; 2.85]	9	2,151	1.77	[1.45; 2.08]	8	2,127	2.30	[1.80; 2.78]
Externalizing problems	50.30	13	4,978	6.95	[4.57; 9.33]	11	2,375	6.48	[3.67; 9.29]	10	2,398	5.71	[3.23; 8.18]
Aggressive behaviors	50.31	10	4,405	4.39	[3.91; 4.86]	9	2,151	4.56	[3.80; 5.30]	8	2,127	3.85	[3.18; 4.51]
Rule-breaking	49.62	9	4,345	1.49	[1.25; 1.72]	8	2091	1.65	[1.33; 1.96]	8	2,127	1.16	[0.88; 1.44]
Thought problems	50.31	10	4,405	1.57	[1.27; 1.86]	9	2,151	1.79	[1.33; 2.24]	8	2,127	1.48	[1.24; 1.72]
Attention problems	50.31	10	4,405	3.26	[2.59; 3.93]	9	2,151	3.95	[3.00; 4.89]	8	2,127	2.87	[2.18; 3.54]
Social problems	50.31	10	4,405	2.10	[1.78; 2.40]	9	2,151	1.87	[1.43; 2.31]	8	2,127	2.16	[1.79; 2.52]
YSR^b^													
Total problems	47.90	8	3,713	45.19	[32.42; 57.95]	4	642	53.31	[43.95; 62.66]	4	560	44.25	[17.86; 70.64]
Internalizing problems	46.97	12	4,123	12.90	[9.73; 16.06]	8	996	10.94	[7.52; 14.36]	9	1,169	14.58	[10.35; 18.80]
Withdrawn/depressed	48.24	8	2,452	5.54	[4.19; 6.88]	8	1,104	3.63	[2.73; 4.51]	8	1,313	4.23	[3.20; 5.24]
Anxious/depressed	45.09	10	3,442	6.75	[5.34; 8.15]	9	1,283	5.46	[3.50; 7.41]	9	1,462	7.24	[4.47; 9.99]
Somatic complaints	44.92	8	2,452	5.31	[3.85; 6.76]	8	1,104	3.31	[2.44; 4.17]	8	1,313	4.37	[3.26; 5.46]
Externalizing problems	47.04	11	3,380	11.31	[9.30; 13.31]	7	612	11.53	[8.71; 14.34]	8	809	11.56	[9.21; 13.89]
Aggressive behaviors	48.24	8	2,452	9.41	[8.18; 10.63]	8	1,104	8.61	[7.81; 9.40]	8	1,313	8.91	[8.24; 9.58]
Delinquent behaviors	47.81	8	2,452	5.36	[4.02; 6.69]	8	1,104	4.59	[3.47; 5.70]	8	1,313	4.07	[3.30; 4.83]
Thought problems	50.05	5	1,566	4.05	[2.88; 5.21]	5	774	3.75	[2.77; 4.73]	5	792	4.20	[2.87; 5.52]
Attention problems	48.15	6	2,228	7.56	[3.84; 11.27]	5	774	5.92	[5.38; 6.45]	5	792	6.41	[5.90; 6.90]
Social problems	50.05	5	1,566	3.10	[2.49; 3.70]	5	774	2.77	[2.33; 3.20]	5	792	3.30	[2.56; 4.03]

Gend, pondered percentage of males. k, number of samples. N, sample size. CI, Confidence Intervals.

**Figure 2:**
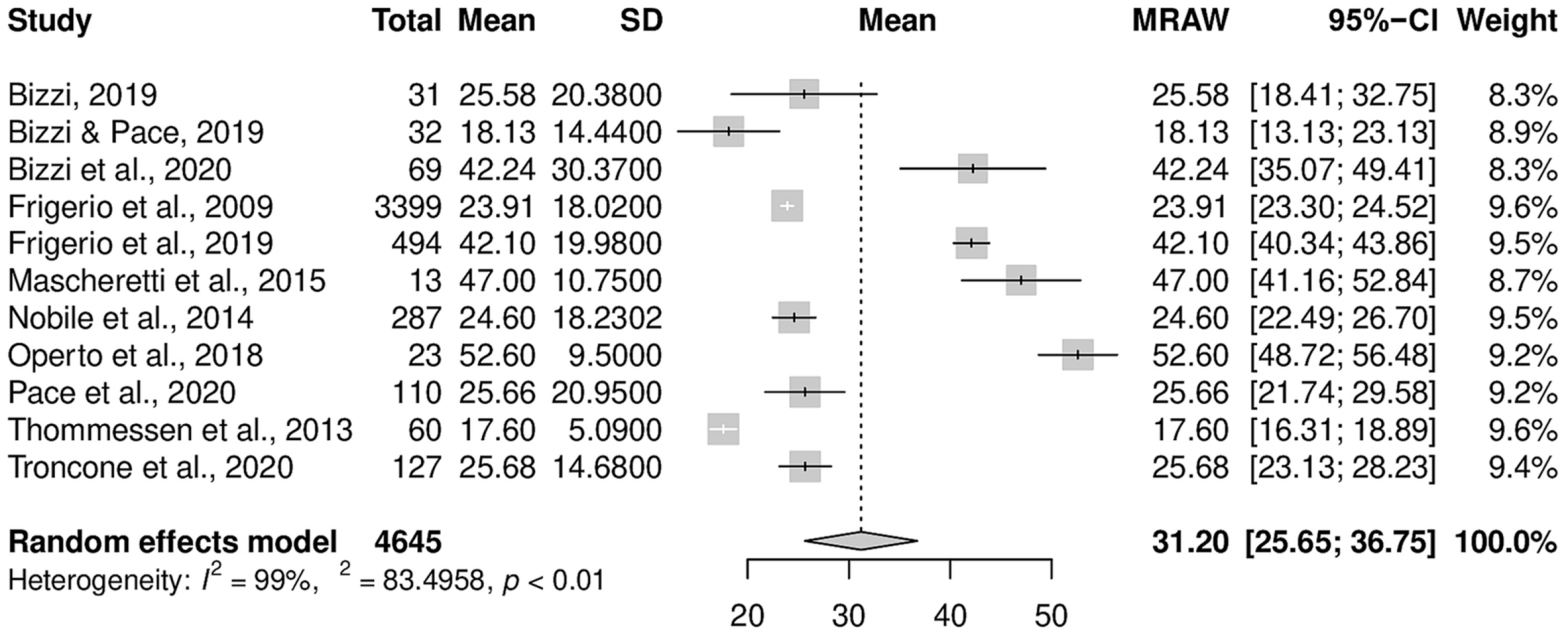
Forest plot for CBCL total scores.

**Figure 3:**
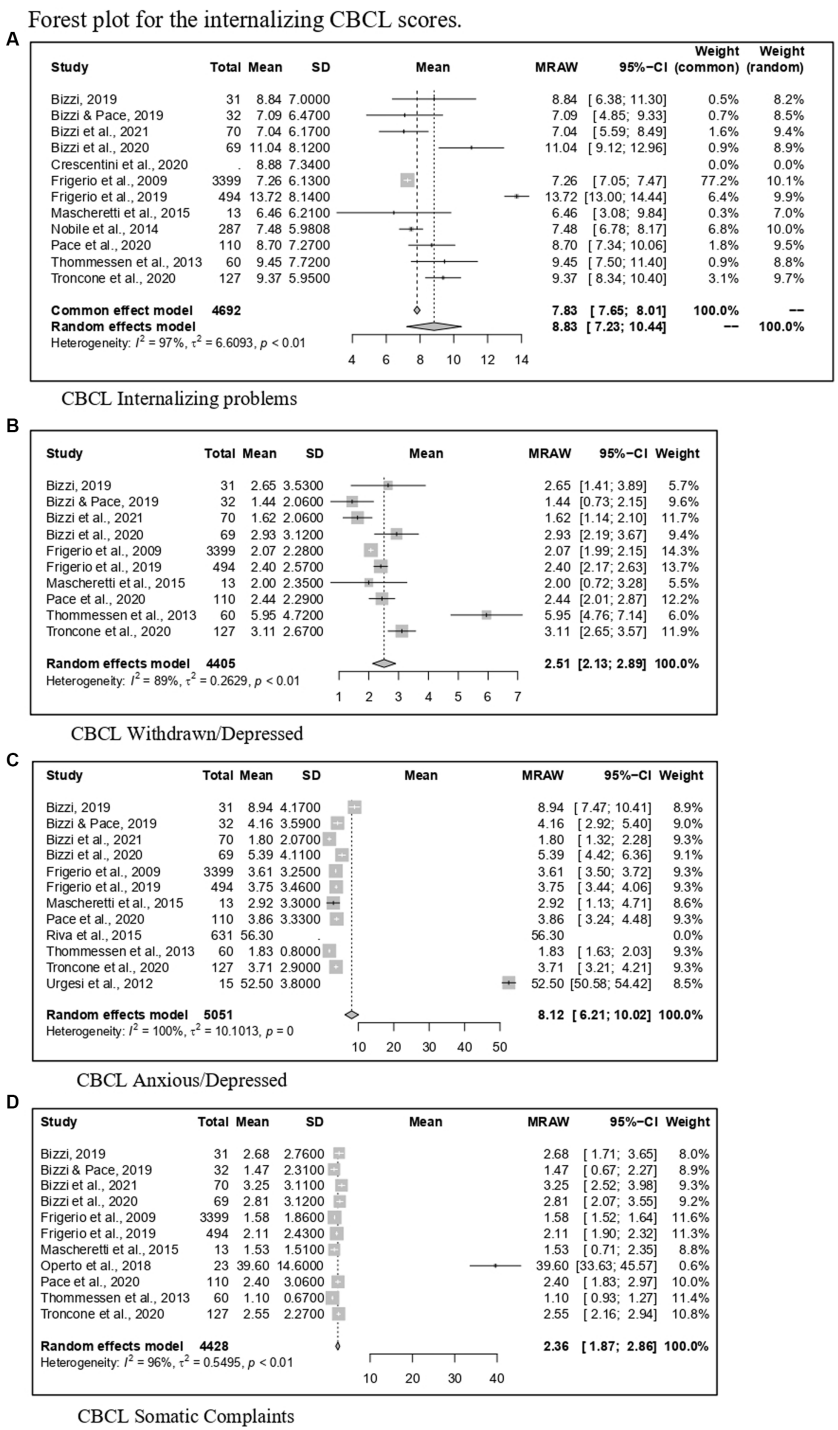
Forest plot for the internalizing CBCL scores. **(A)** CBCL internalizing problems. **(B)** CBCL withdrawn/depressed. **(C)** CBCL anxious/depressed. **(D)** CBCL somatic complaints.

**Figure 4:**
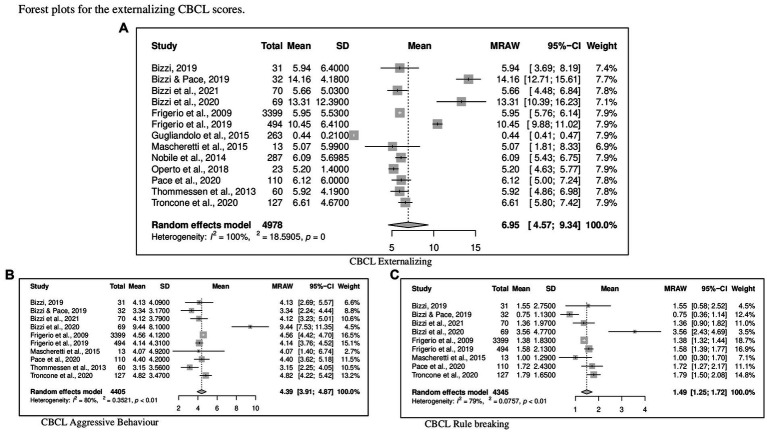
Forest plot for the externalizing CBCL scores. **(A)** CBCL externalizing. **(B)** CBCL aggressive behavior. **(C)** CBCL rule breaking.

**Figure 5:**
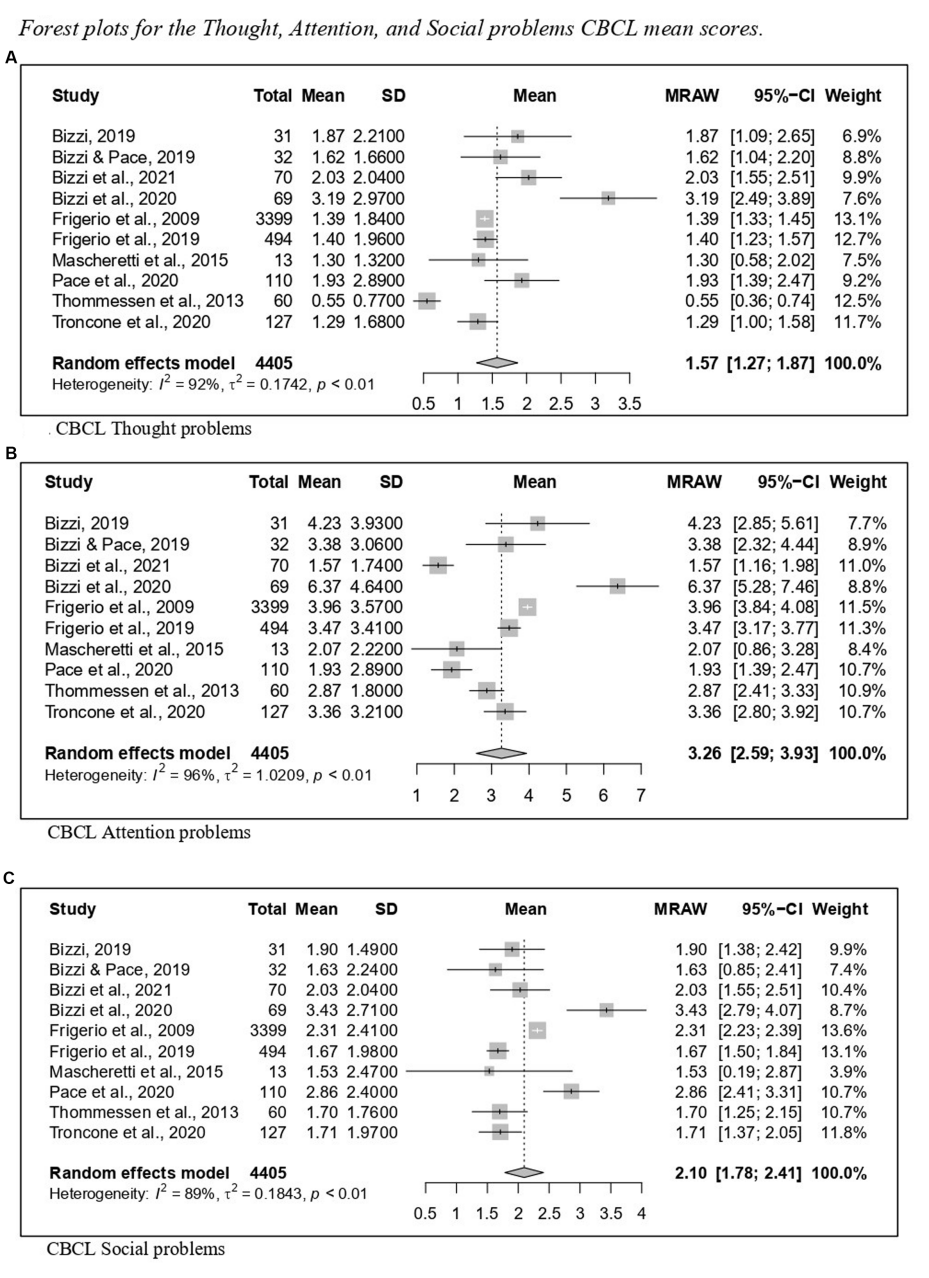
Forest plot for the thought, attention, and social problems CBCL mean scores. **(A)** CBCL thought problems. **(B)** CBCL attention problems. **(C)** CBCL social problems.

Regarding CBCL, Figure 2 reports the forest plot resulting from data of Total problems, Figures 3, 4 show those for Internalizing problems (and related subscales) and for Externalizing problems (related subscales) respectively. Figure 5 displays those for Thought, Attention and Social problems scales.

**Figure 6:**
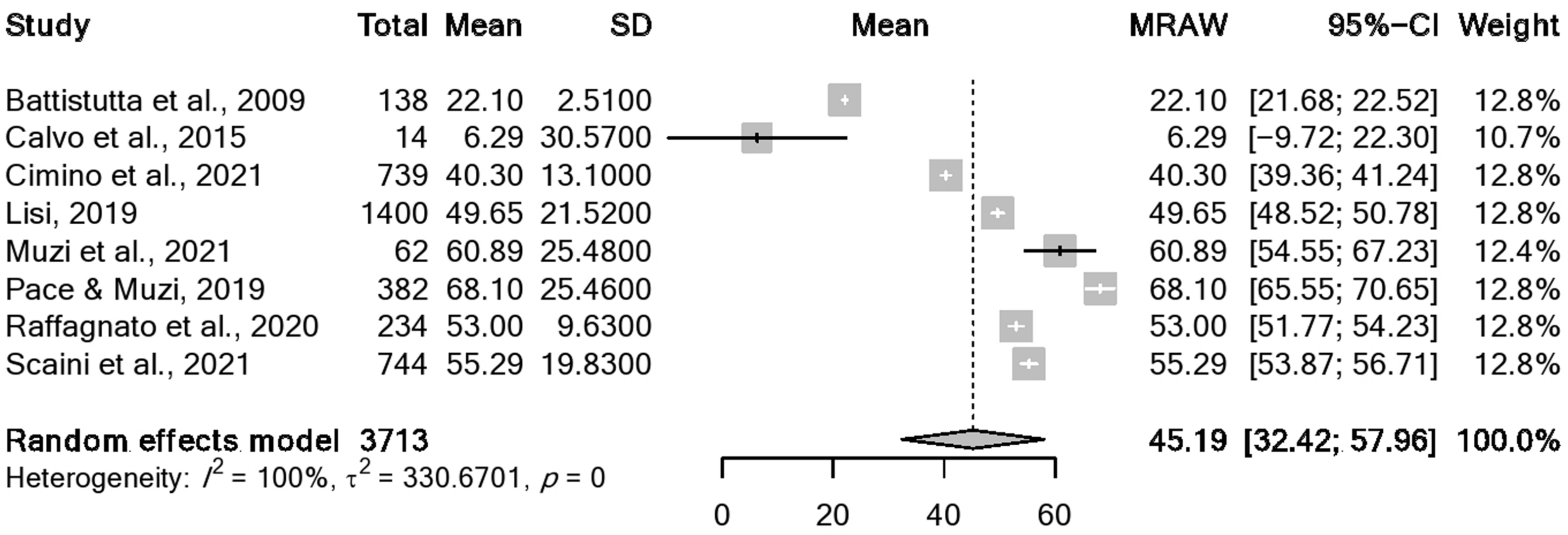
Forest plot for the total YSR scores.

**Figure 7:**
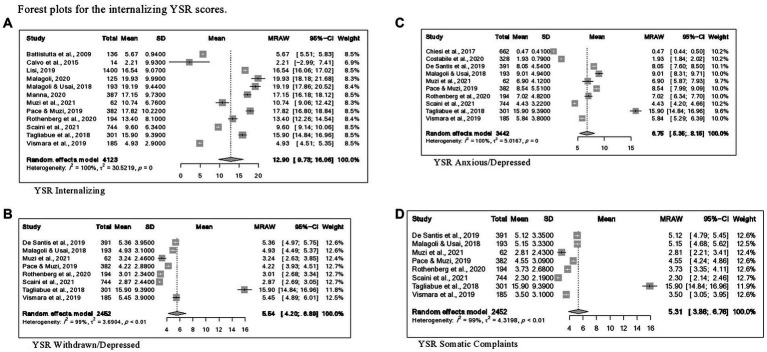
Forest plots for the internalizing YSR scores. **(A)** YSR internalizing. **(B)** YSR withdrawn/depressed. **(B)** YSR anxious/depressed. **(B)** YSR somatic complaints.

**Figure 8:**
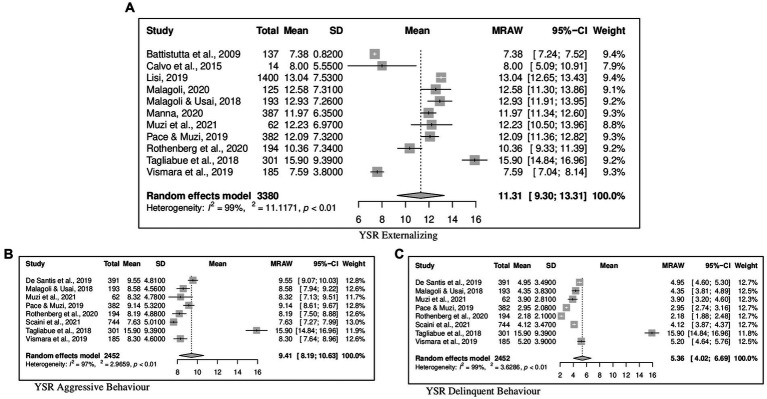
Forest plot for the externalizing YSR scores. **(A)** YSR externalizing. **(B)** YSR aggressive behavior. **(C)** YSR delinquent behavior.

**Figure 9:**
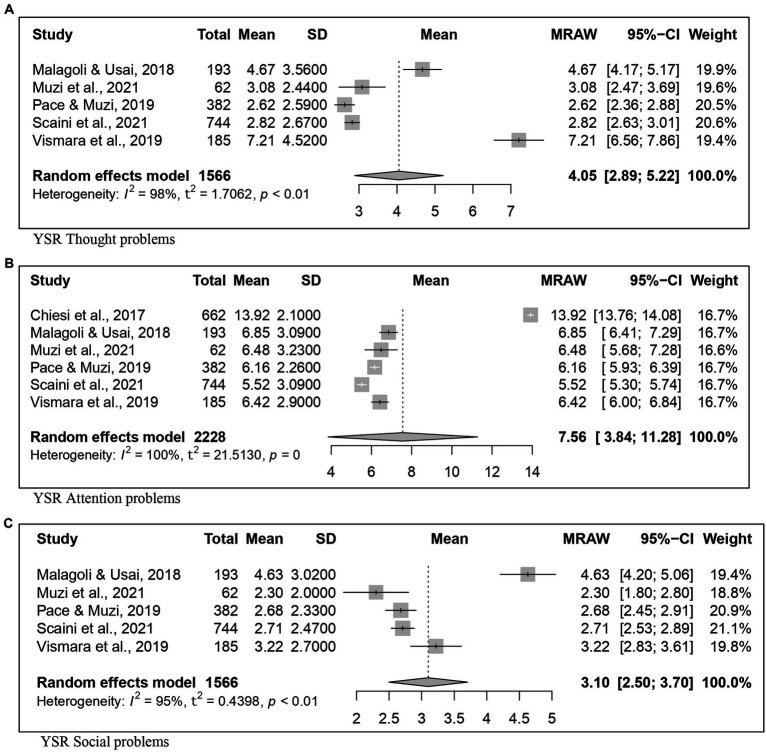
Forest plot for the thought, attention, and social problems YSR scores. **(A)** YSR thought problems. **(B)** YSR Attention problems. **(C)** YSR social problems.

Concerning the YSR results, forest plots of Total problems, Internalizing problems, and Externalizing problems with related subscales are reported in Figures 6–8 respectively. Figure 9 shows those for Thought, Attention and Social problems scales.

Appendices B, C contain funnel plots regarding pooled means in the CBCL and the YSR. Results of the Egger tests were never significant except for the scores obtained on the Anxiety dimension of the YSR. Therefore, Trim and Fill was applied, resulting in a corrected pooled mean equal to 1.34 (see Appendix D).

Also, sensitivity analyses removing studies with small sample sizes were conducted (detailed results are displayed in Appendix D in the “CBCL small” and “YSR small” sections). Regarding the CBCL data, the pooled mean as well as the heterogeneity index largely dropped resulting to be 3.93 and 360.40, respectively. Also, regarding the Somatic CBCL dimensions, it was observed that removing studies with small sample sizes greatly reduced heterogeneity (54.46% of reduction). These analyses were carried out on a few dimensions of the YSR because of the limited number of studies with small sample sizes. No significant increase or reduction of indexes was observed.

### RQ2: moderation of gender and age

Only one significant moderation effect was found on the CBCL pooled means. Specifically, the percentage of males in the samples negatively moderated the Anxious/Depressed pooled mean (*Q* = 12.56, *p* < 0.05, *ß* = −0.48, *se* = 13), which means that the pooled mean of Anxious/Depressed scale decreased along with the increase of the proportion of males in the sample. Table 2 displays the pondered percentage of males for each of the pool of contributions considered in the moderation analyses. As shown in Table 2, the Anxious/Depressed CBCL dimension has the most unbalanced composition regarding gender.

The remaining moderation effects illustrated subsequently were all on YSR pooled means. There was no effect due to the gender composition, while there were several significant moderating effects due to age. Specifically, in the whole mixed-gender sample, as mean age of the samples increased, the Total problems mean increased (*Q* = 61.11; *p* < 0.05; *ß* = 10.80; *se* = 4.49) and the Attention problems mean decreased (*Q* = 19.65; *p* < 0.05; *ß* = −3.81; *se* = 0.86). Moreover, as mean age increased, the Anxious/Depressed mean obtained by males decreased (*Q* = 9.07; *p* < 0.05; *ß* = −3.17; *se* = 1.05). The remaining non-significant results are all displayed in Appendix D.

### RQ3: moderation of the studies’ variables pre-post pandemic, publication year, and quality

As stated above, the excessive homogeneity of data (being post-pandemic only one study was carried out with the CBCL and three with the YSR) did not allow to perform a moderation analysis using the period of data collection as a categorical moderator. Instead, the role of these variables was explored through sensitivity analyses.

Because no study using the CBCL was conducted during the post-pandemic period, these analyses were not performed for this outcome. Regarding the YSR pooled mean, it was observed that, when removing studies conducted in the post-pandemic period, heterogeneity was reduced by 25% in several subscales. These include Aggression, Withdraw, Anxiety, Attention, and Somatic Problems. Also, pooled means greatly increased on the Withdraw and Somatic subscales. Detailed findings are available in the “YSR pandemic” section of Appendix D.

The same approach was adopted to explore changes in pooled mean and heterogeneity when removing studies without a cross-sectional design of research. This was tested when at least one study adopted a not cross-sectional design of research. Regarding the CBCL, pooled mean never significantly changed. However, we observed that heterogeneity was reduced by nearly 50% in the case of Internalizing, Externalizing, Attention, and Somatic problems scales. The same effect was found regarding the Aggression, Rule, Withdraw, and Somatic problems of the YSR. In addition, removing studies without a cross-sectional design led to a reduction of nearly 25% of the pooled means estimated on the Rule, Withdraw, and Somatic subscales of the YSR. All results are displayed in the “CBCL design” and “YSR design” of Appendix D.

Then, moderation analyses were carried out using the publication year as a continuous moderator. There were no significant moderation effects of any variables on CBCL pooled means (see Appendix C).

Concerning moderation effects on YSR pooled means in the whole sample, as the publication year increased (i.e., more recent publication), scores increased on the total (*Q* = 6.22; *p* < 0.05; *ß* = −3.41; *se* = 1.37) and externalizing problems (*Q* = 8.82; *p* < 0.05; *ß* = −0.39; *se* = 0.13), while a reverse effect was observed regarding the mean scores of Withdrawn (*Q* = 5.98; *p* < 0.05; *ß* = 1.39; *se* = 0.57), Attentional (*Q* = 4.64; *p* < 0.05; *ß* = 1.51; *se* = 0.07) and Somatic complaints (*Q* = 6.86; *p* < 0.05; *ß* = 1.48; *se* = 0.56) narrow-band scales.

Also, as the publication year increased, the total problems in the only-males sample decreased (*Q* = 4.71; *p* < 0.05; *ß* = −4.46; *se* = 2.06) and the Somatic complaints of the only-females group increased (*Q* = 5.08; *p* < 0.05; *ß* = 1.13; *se* = 0.50).

Lastly, as the methodological *quality* of the study increased, the Total problems mean score increased (*Q* = 5.27; *p* < 0.05; *ß* = 18.75; *se* = 8.17) and the Somatic complaints decreased (*Q* = 7.01; *p* < 0.05; *ß* = −2.61; *se* = 0.98) in the whole sample, as well as the Internalizing problems mean of the only-males group (*Q* = 4.35; *p* < 0.05; *ß* = −2.71; *se* = 1.30).

### RQ4: major trends of studies on the relationships between emotional-behavioral problems and other outcomes

As shown in Table 1, 27 of the 44 studies (61.4%) included in the systematic review explored the difficulties assessed with the ASEBA questionnaires together with other outcomes. A narrative description of the findings of these studies is reported in Table 1. There are three major trends identified in the current literature: The larger part of these studies (*n* = 8, 29.6%) investigated adolescents’ problems in respect to attachment (42, 44, 45, 52, 54, 64, 67, 72); a second trend investigated problems and other comorbid symptoms (*n* = 8, 29.6%), i.e., internet or social media misuse (48, 50, 57, 67), eating disorders (71, 81), alcohol misuse (57), sleep problems (66). A last identifiable trend focused the role of parental features (*n* = 5, 18.5%) such as parenting style/control (61, 79), patterns of communication (60), and symptoms (69, 83).

The rest of studies investigated relationships with miscellaneous variables. Particularly, these are various psychological features such as mindfulness skills (49, 70), emotion dysregulation (62, 75), psychological inflexibility (70, 77), autistic traits (46), reflective functioning (44), verbal skills (44), and perceived self-efficacy (50), while two studies investigated problems’ together with various dimensions of school engagement (51, 58).

## Discussion

This study reviewed data of the ASEBA questionnaires CBCL, YSR, and TRF in the versions of the year 2001 in Italian adolescents. The aims were to review studies on emotional-behavioral difficulties of Italian adolescents and to investigate the moderating role played by sociodemographic factors, time of assessment, and quality of studies. This first part is on community samples, in order to provide an updated picture of mental health of Italian adolescents with typical functioning and no clinical or at-risk conditions.

A preliminary consideration is that none of the included Italian studies considered the Teacher Report Form, which makes these results poorly informative for practitioners wishing to employ this questionnaire. This absence should not be interpreted as a complete lack of use or interest in TRF among Italian practitioners, as perhaps Italian studies that include this questionnaire did not respond to inclusion criteria, e.g., lower age range, or data not retrieved. In addition, the TRF version mainly used with community sample could be the one updated in 1991 (18), which was excluded in this study. Therefore, enlarging the research by including all versions of the ASEBA measures could help clarify this absence. However, the results of this review can be of interest to all practitioners employing the parent-report CBCL and the adolescent-report YSR.

### Means of emotional-behavioral problems in Italian adolescents

Results answering the first research question reveal a different picture than the one offered by previous Italian epidemiological studies and cross-cultural comparisons (14, 18, 21, 84).

The Total problems pooled mean in the CBCL 6–18 was eight points higher than the one registered with the CBCL 4–18 in Italian adolescents aged 12–18 years (13). Thus, the mean reported in this review resulted in 6–7 points higher than the pooled intercountry one in Rescorla et al. (18), contrary to the previously registered Italian mean, which is slightly below the international average (18). In addition, the mean of total problems as self-reported by Italian adolescents in the YSR was 4 points lower here than previously (14), but still in the highest positions of the international rank (18). Therefore, Italian adolescents’ pooled means of Total problems differed from those resulting from previous versions of the instruments.

On the one hand, differences between the pooled means calculated here and previous normative results are merely descriptive, with no analyses having been conducted to test their statistical significance. Thus, a future contribution wanting to address this issue could calculate pooled means obtained by adolescents on the past and current versions of the ASEBA instruments to test the moderating role of the version used.

On the other hand, albeit descriptive, these differences between results from 2001 and previous ASEBA instruments solicit reflection and different possible explanations. First, an explication is suggested by Rescorla et al. (18, 85) observation of cultural differences in rating difficulties, which may suggest attributing these differences to changes in the Italian culture that have occurred over time. In this circumstance, Italian parents could be more prone to perceive difficulties in their children. Of note, this explanation is partially supported by the results of analyses performed here, which examine the role of time in the pool of contributions included. This is fully commented on in the next paragraphs. Second, these differences may be due to discrepancies between the versions of the instruments. For this, we invite researchers to be cautious when comparing scores obtained with different versions of the same ASEBA instrument. However, this explanation may be insufficient as few items have been modified from the past versions of the questionnaires. Third, there could be differences between the Italian adolescents recruited for the epidemiological normative studies (13, 14) and those participating in the studies included in the current review. For instance, this work did not check the role of some potentially confounding variables, such as age and gender, on the differences observed between normative and this review’s pooled means.

### Gender and age differences

A traditional line of investigation concerns gender and age differences in ASEBA rates (18, 86), the object of the second research question in this review.

#### Participants gender

Results regarding the moderating role of gender composition were unexpected. Indeed, most studies using CBCL or YSR and that compared scores of girls and boys found significant differences. For instance, a previous Italian contribution (13) found that parents assigned higher scores to boys, compared to girls, on many scales except for more somatic complaints. In addition, the international literature often reported more total and externalizing problems in males and more internalizing problems in females (18), mirroring wider epidemiological data (15, 87). Instead, the gender moderated only Anxiety mean scores of the CBCL, where a higher percentage of girls in the samples corresponded to higher levels of Anxiety/Depression, in line with previous studies (18). For the remaining data, contrary to hypotheses and literature, there was no moderating role of gender on mean scores obtained on all the other ASEBA scales. These non-significant results may have several explanations. First, this outcome could be due to poor heterogeneity in the gender composition. Indeed, the pooled percentage of males and females was almost equal to 50% for most dimensions, except for anxiety/depression where females were slightly overrepresented, and a difference was found indeed. Second, as the age increased, anxiety decreased in males, suggesting a moderation role of gender in symptoms found in other studies (13, 18). Therefore, a future study could aim to perform a meta-analysis on gender differences observed in studies using the ASEBA to better address the issue of the identification of differences between girls and boys.

#### Participants age

In this review, age was not influential on the CBCL 6–18 scores, while international studies found that parents of older teenagers tend to assign higher scores on most scales (18). This discrepancy in results could be explained by a cultural dissimilarity or by differences between the two versions of the questionnaire, which should be further investigated. In addition, changes may have occurred in the last decade in parents’ assessment of difficulties in their teenagers. The explanation for this could be found in a cultural transition prompted by media, which has made parents more aware, and therefore more prepared, of problematic behaviors in their teenage children since middle school, regardless of their gender (88).

Instead, results obtained with the self-report YSR were more in line with the literature (18), confirming that older teenagers tend to report more total problems, more attention problems and lower anxiety than younger ones. In absence of previously published Italian data on YSR, these results are difficult to discuss in the Italian context, but they seem to suggest a certain homogeneity in the way Italian teenagers evaluate their difficulties. This could be ascertained with future dedicated studies.

### Effects of studies’ characteristics

For the third research question, the effects of some studies’ characteristics on scores were examined.

#### Pre/post-pandemic studies

In light of recent literature findings suggesting an increase in emotional-behavioral difficulties after the COVID-19 pandemic (6), although not completely supported by Italian literature (89), this review aimed to analyze differences in studies published pre- or post-pandemic. Unfortunately, given that only four studies out of 34 were published post-pandemic (50, 53, 64, 67), it was impossible to carry out analyses helpful to increase the knowledge about the effect of the pandemic. This calls for more Italian research on the topic employing the ASEBA questionnaires so that future updates of this meta-analysis could respond to this part of the research question.

#### Publication year

Results partially confirm the literature reporting an increase in emotional-behavioral difficulties among teenagers over the last decades (5, 90). In contrast with previous research, no growth was revealed when informants were parents, and a general decrease in total problems and externalizing problems was observed when the inquired person was a teenager. On the other side, internalizing problems increased over years in line with the literature (9), but in form of depressive symptoms and somatic complaints rather than anxiety. This suggests a change in the form of expression of the illness which should be further investigated. An additional observation converging with previous studies consists in the increase of attentional problems, potentially due to a growth in screen use by children and adolescents in the last decades, particularly on mobile phones (91). Of note, even if results are not completely in line with worldwide epidemiological investigation, which reports an increase in more syndromes (91), these trends align with those observed in Northern-European countries (5). This pattern of results suggests framing the conclusions of the ASEBA Italian study conducted among adolescents within the European literature rather than the worldwide one. This should be done considering the peculiarities of European Countries in difficulties distribution and rating (87, 90).

#### Quality of studies

In line with the PRISMA 2020 guidelines and recent attention to the quality of the studies included in reviews, especially when meta-analytical findings were brought (36), this study includes a check of the effect of studies’ quality on results. These revealed that teenagers were rated with higher scores in studies of higher quality, except for the somatic complaints, which were less in higher-quality works. Although this evaluation may be affected by those who conduct it, it seems relevant that the scores on some scales vary according to the quality of the study. This is because it can indicate weaknesses and future lines of investigation on the goodness of the instrument, or in the research process. In this review, the included contributions obtained low to medium scores of quality (from 1 to 4 in a range 1–7), and funnel plots revealed a degree of study heterogeneity. This suggests improving the quality of Italian research with the ASEBA questionnaires. For instance, authors should improve the clarity and completeness of the study method and results reporting during the paper writing. In general, the conspicuous body of international methodological research on the ASEBA system seems to have poorly investigated the impact of the quality of the studies on outcomes, suggesting the implementation of research in this direction.

### Trends of research on ASEBA problems and psychological/psychopathological outcomes

Results for the fourth research question highlighted that most of studies investigated relationships between adolescents’ emotional-behavioral problems and other outcomes, suggesting this as an interest of Italian researchers. The scope of this systematic review was to map major trends, and the included studies allowed to detect three major trends. Specifically, Italian contributions included in the systematic review seem to pay similar attention to the relationships of emotional-behavioral problems and both attachment and comorbid symptoms, following the international trends (18, 91, 92). In particular, Italian researchers employed the ASEBA questionnaires to check the effect of attachment insecurity (42, 44, 45, 52, 54, 64, 67, 72) and to explore the relationships between emotional-behavioral problems and comorbid symptoms of internet addiction and eating disorders (48, 50, 57, 67, 71, 81). This is in line with an international trend (93). Still, Italian studies contributed to community-based investigation on the role of parental features and other psychological aspects (60, 61, 69, 79, 83). Overall, Italian research trends that consider the ASEBA questionnaires follow organizational and research recommendations to design empirically grounded prevention (4, 18, 84, 94). Some of them also follow the Rescorla et al. (85) suggestions to go beyond the exclusive focus on internalizing and externalizing broad-band scales. In addition, they suggest to implement research on narrow-band scales (42, 49, 54, 67), and to include research on relationships between children problems and parental features (60, 61, 69, 79, 83). However, these results only aimed to be descriptive, and future international meta-analyses would address these topics to provide and empirically supported based to findings and suggestions of single studies.

## Conclusion and implications for clinical practice

In sum, this systematic review investigates emotional-behavioral difficulties in Italian adolescents aged 11–18 years. The studies provide researchers and practitioners with pooled data which are useful to frame their findings with the ASEBA measures in the Italian context, and that are potentially helpful for cross-cultural comparisons.

First, the few differences related to gender and age suggest approaching males and females, regardless of age, with no expectations of the type of symptoms they might exhibit. At best, results on trends might suggest paying particular attention to the presence of depressive symptoms and somatic complaints, or attentional symptoms potentially prodromal of ADHD. However, this consideration should be accepted with great caution because this review mainly includes pre-pandemic studies, and it could therefore mainly draw the picture of Italian adolescents before the disease’s outbreak. Given that the literature suggests an increase in anxiety during the COVID-19 widespread that could not be substantiated due to a lack of contributions, more post-pandemic studies are expected for a more complete picture (6).

Further, given the comorbidities and associations reported in this review, practitioners detecting emotional-behavioral difficulties in community adolescents should be recommended to also screen for eating disorders and social media addiction subthreshold symptoms. This could be useful to perform more comprehensive prevention. In this regard, the results of this review may support the utility of using the ASEBA questionnaires to detect emotional-behavioral symptoms in community adolescents for preventive purposes, for example through school surveys.

Lastly, although this review did not investigate adolescent-parent agreement, discrepancies in scores suggest to practitioners to take into consideration the recommendation from the international ASEBA literature (84, 95). This refers to the use a multi-informant approach when possible. The aim is to depict a more precise picture of the adolescent’s situation. This is also done by reducing the probability of bias resulting from an underestimation of the problems by parents and an overestimation of the same by their teenage children. In this regard, although this review could not include data on TRF, practitioners wanting to use this teacher-report questionnaire should consider that parent and teachers’ ratings show discrepancies as well (95).

### Limitations and future lines of research

As the first effort to synthesize data with the ASEBA questionnaires in Italy, this review has the strength to provide provisional parameters to contextualize single results in an Italian context. However, it also has many limitations that urge caution in the reliance on its results. First, the number of the included studies is limited, especially considering that 38 contributions were potentially eligible but it was impossible to isolate data on participants aged 11–18 years or on the scales. In addition, 10 contributions were excluded by the meta-analysis as necessary data could not be retrieved. Therefore, future Italian studies with ASEBA questionnaires should include means to contribute to this epidemiological effort. Moreover, the studies considered were of low to medium quality, limiting the soundness of our conclusions. Second, none of the retrieved articles employed the TRF, so this review could not provide synthetical data on it. This solicits more research with this questionnaire to collect data on adolescents’ problems at school, necessary to complete a picture of the mental health of the community Italian teenagers within and outside the family. For instance, this may hinder the advances of knowledge in specific fields of study such as distress expressed and experienced in the school’s context such as perpetration and victimization of bullying or social anxiety experienced with peers (96). Third, southern-east areas of the countries appear underrepresented, calling for more studies for more a comprehensive examination. In this regard, given that most contributions have local coverage or at best are multicentric in two or three cities, Italian researchers and institutions are called to a joint effort to coordinate epidemiological research with national reach. Further, the paucity of post-pandemic studies hindered the possibility to investigate changes in Italian adolescents’ mental health after the COVID pandemic, soliciting more publications on this topic.

Then, the conclusions we have drawn regarding emotional and behavioral problems in the population of Italian adolescents could not be fully appreciated without considering the clinical population. For instance, interesting studies highlighted the role of emotional and behavioral problems in vulnerable populations of adolescents with autism spectrum disorder (97) as well as the interplay of these problems with psychopathological variables typically involved in mental disorders (98). In this regard, the second part of this study, consisting of replicating this study on clinical samples, may be precious to better contextualize the data discussed here.

Moreover, we should note that the lack of significant results regarding the moderating role of gender may be accounted for by the lack of consideration of other potential confounding variables. For instance, non-binary gender and some cultural aspects such as religion have been showed to impact psychological outcomes in Italian adolescents (99, 100). From this perspective, these not documented variables may have introduced heterogeneity in our data and confounded the role of gender.

In addition, some choices applied to the search strategy may have limited the exhaustivity of the search. First, the use of age filters may have limited the number of records detectable and then retrieved. Second, the search for gray literature on Google Scholar should have been combined with other strategies as suggested by some authors (30, 101). Third, the choice of using quoted terms and not the MeSH term for “Italy” in PubMed may have affected the research results.

Another limitation is that this review did not investigate differences and discrepancies between CBCL and YSR. These emerged not only in means but also in moderation analyses, where several effects were found only on the YSR, e.g., age, publication year, and quality. In general, the YSR has been the object of fewer methodological studies compared to the other two measures. For this reason, future research should implement efforts to focus strengths and limits of this questionnaire, particularly in Italy where it appeared to be the most used in the included contributions.

## Data availability statement

The original contributions presented in the study are included in the article/Supplementary material, further inquiries can be directed to the corresponding author.

## Author contributions

CP: conceptualization, methodology, project administration, supervision, data curation, visualization, validation, writing – original draft, and writing – review and editing. SM: conceptualization, methodology, investigation, data curation, visualization, writing – original draft, and writing – review and editing. AF: visualization, validation, and writing – review and editing. VB: screening, investigation, and writing – review and editing. GR: methodology, project administration, supervision, investigation, data curation, visualization, validation, writing – original draft, and writing – review and editing. All authors contributed to the article and approved the submitted version.
