# Supplementary material for: Twenty years of emotional-behavioral problems of community adolescents living in Italy measured through the Achenbach system of empirically based assessment (ASEBA): a systematic review and meta-analysis
Source: Front Psychiatry. 2023 Dec 11;14:1161917. doi: 10.3389/fpsyt.2023.1161917 (PMC10749347; doi:10.3389/fpsyt.2023.1161917)
Supplement: Supplementary file 2 [file Data_Sheet_1.DOCX]

1. CBCL Total problems

2. CBCL Internalizing problems

3. CBCL Withdrawn/depressed

4. CBCL Anxious/depressed

5. CBCL Somatic complaints

6. CBCL Externalizing problems

7. CBCL Aggressive behaviors

8. CBCL Rule breaking

9. CBCL Thought problems

10. CBCL Attention problems

11. CBCL Social problems
