# Supplementary material for: Twenty years of emotional-behavioral problems of community adolescents living in Italy measured through the Achenbach system of empirically based assessment (ASEBA): a systematic review and meta-analysis
Source: Front Psychiatry. 2023 Dec 11;14:1161917. doi: 10.3389/fpsyt.2023.1161917 (PMC10749347; doi:10.3389/fpsyt.2023.1161917)
Supplement: Supplementary file 3 [file Data_Sheet_2.DOCX]

**Appendix A. Syntax used on all databases for systematic search**

**SCOPUS:**
 ( ALL ( "Child Behavior Checklist" OR "CBCL 6-18" OR "Youth Self Report" OR "YSR" OR "Teacher Report Form" OR "TRF" OR "Questionario sul comportamento del giovane" OR "Achenbach System of Empirically Based Assessment" OR "ASEBA" OR " Questionario sul comportamento del bambino" ) AND ALL ( "italian" OR "italy" OR "italiani" OR "italiana" OR "italiano" OR "Italia" ) ) AND PUBYEAR > 2000 AND PUBYEAR < 2022 AND ( LIMIT-TO ( DOCTYPE , "ar" ) OR LIMIT-TO ( DOCTYPE , "ch" ) OR LIMIT-TO ( DOCTYPE , "cp" ) OR LIMIT-TO ( DOCTYPE , "bk" ) )

**EBSCO, including PsycInfo, PsycArticles and Behavioral Science Collection:**
( "child behavior checklist for ages 6-18" OR "CBCL 6-18" OR "Youth Self Report" OR "YSR" OR "Teacher Report Form" OR "TRF" OR "Questionario sul comportamento del giovane" OR "Achenbach System of Empirically Based Assessment" OR "ASEBA" OR " Questionario sul comportamento del bambino" ) AND ( "italian" OR "italy" OR "italiani" OR "italiana" OR "italiano" OR "Italia" )
FILTER AGE GROUPS (school age, adolescence, young adulthood); published 2001 to 2021

**PUBMED:**
("Child Behavior Checklist" OR "CBCL 6-18" OR "Youth Self Report" OR "YSR" OR "Teacher Report Form" OR "TRF" OR "Questionario sul comportamento del giovane" OR "Achenbach System of Empirically Based Assessment" OR "ASEBA" OR " Questionario sul comportamento del bambino") AND ("italian" OR "italy" OR "italiani" OR "italiana" OR "italiano" OR "Italia")
*Applied filters*: from 2001 to 2021; Child 6-12 years; Adolescent 13-18 years

**WEB OF SCIENCE^[[1]](#footnote-1)^:**
(TS = ("Child Behavior Checklist 6-18" OR "CBCL" OR "Youth Self Report" OR "YSR" OR "Teacher Report Form" OR "TRF" OR "Questionario sul comportamento del giovane" OR "Achenbach System of Empirically Based Assessment" OR "ASEBA" OR " Questionario sul comportamento del bambino")) AND (TS = ("italian" OR "italy" OR "italiani" OR "italiana" OR "italiano" OR "Italia"))
Timespan: 2001-2021. Indexes: SCI-EXPANDED, SSCI, A&HCI, CPCI-S, CPCI-SSH, BKCI-S, BKCI-SSH, ESCI.

**PROQUEST^[[2]](#footnote-2)^**:
( "child behavior checklist for ages 6-18" OR "CBCL" OR "Youth Self Report" OR "YSR" OR "Teacher Report Form" OR "TRF" OR "Questionario sul comportamento del giovane" OR "Achenbach System of Empirically Based Assessment" OR "ASEBA" OR " Questionario sul comportamento del bambino" ) AND ( "italian" OR "italy" OR "italiani" OR "italiana" OR "italiano" OR "Italia" )
*Applied filters*: 2001 to 2021; children & youth OR teenagers OR children OR adolescents OR adolescent OR child; Article OR Dissertation/Thesis OR Evidence Based Healthcare OR Report OR Undefined OR Working Paper/Pre-Print OR Conference

**GOOGLE SCHOLAR**

( "child behavior checklist for ages 6-18" OR "CBCL 6-18" OR "Youth Self Report" OR "YSR" OR "Teacher Report Form" OR "TRF 6-18" OR "ASEBA" ) AND ( "italian" OR "italy" OR "italiani" OR "italiana" OR "italiano" OR "Italia" )

1. Database searched: BioProject, Books, ClinVar, Conserved Domains, dbGaP, Gene, GEO DataSets, GEO Profiles, HomoloGene, MedGen, Nucleotide, OMIM, PopSet, Protein, PubChem BioAssay, PubChem Compound, PubChem Substance, PubMed, SNP, SRA, Structure, Taxonomy. [↑](#footnote-ref-1)
2. Database searched: ProQuest Database 12 [↑](#footnote-ref-2)
