# Supplementary material for: Twenty years of emotional-behavioral problems of community adolescents living in Italy measured through the Achenbach system of empirically based assessment (ASEBA): a systematic review and meta-analysis
Source: Front Psychiatry. 2023 Dec 11;14:1161917. doi: 10.3389/fpsyt.2023.1161917 (PMC10749347; doi:10.3389/fpsyt.2023.1161917)
Supplement: Supplementary file 4 [file Data_Sheet_3.DOCX]

1. YSR Total problems

2. YSR Internalizing problems

3. YSR Withdrawn/depressed

4. YSR Anxious/depressed

5. YSR Somatic complaints

6. YSR Externalizing problems

7. YSR Aggressive behaviors

8. YSR Delinquent behaviors

9. YSR Thought problems

10. YSR Attention problems

11. YSR Social problems
